# Daily, Weekly, Seasonal, Within- and Between-individual Variation in Nutrient Intake According to Four Season Consecutive 7 Day Weighed Diet Records in Japanese Female Dietitians

**DOI:** 10.2188/jea.12.85

**Published:** 2007-11-30

**Authors:** Yuko Tokudome, Nahomi Imaeda, Teruo Nagaya, Masato Ikeda, Nakako Fujiwara, Juichi Sato, Kiyonori Kuriki, Shogo Kikuchi, Shinzo Maki, Shinkan Tokudome

**Affiliations:** 1 Nagoya-Bunri College, Nishi-ku, Nagoya.; 2 Nagoya City School of Nutrition, Mizuho-ku, Nagoya.; 3 Nagoya City University Medical School, Mizuho-ku, Nagoya.; 4 University of Occupational and Environmental Health, Yahatanishi-ku, Kitakyushu.; 5 Nagoya City University School of Nursing, Mizuho-ku, Nagoya.; 6 Nagoya University Hospital, Showa-ku, Nagoya.; 7 Aichi Medical University School of Medicine, Nagakute-cho, Aichi-gun.; 8 Aichi Prefectural Dietetic Association, Kita-ku, Nagoya, Japan.

**Keywords:** daily, weekly and seasonal variance, Japanese female dietitians, nutrient intake, weighed diet records, within- and between-individual variation

## Abstract

Objective: To study daily, weekly, seasonal, within- and between-individual variance in intake of selected nutrients and minimal days necessary for assessing true intake with a specified degree of error based on four season consecutive 7 day weighed diet records (WDRs). Subjects and Methods: We evaluated consumption of energy and 30 nutrients based on four season consecutive 7 day WDRs from 80 Japanese female dietitians in 1996-1997. We examined daily, weekly, seasonal, within- and between-individual variation in nutrient intake, relative contributions of their variances to total variance, and minimal days required to estimate a person’s nutrient intake within 10% and 20% of their true mean with 95% confidence intervals. Results: The relative contributions of variation for all nutrients by person were larger than those by day, week and season. Within-individual variances were greater than the between-individual variances. The ratios of within- vs. between-individual variances thus ranged from 1.3 - 26.9. Minimal days necessary for estimating nutrient consumption per person within 10% (20%) of the true mean with 95% confidence intervals ranged from 10-35 (3-9) days for energy and major nutrients and 15-640 (4-160) days for micro-nutrients. Conclusions: The relative contributions of variability by person were largest for all nutrients, followed by those due to sequence of days, season and day of week. Within-individual variation was greater than between-individual variation. Minimal days necessary for ascertaining major nutrients were in general fewer than micro-nutrients.

## INTRODUCTION

There are several approaches for estimating consumption of nutrients, which include diet records/weighed diet records (abbreviated DRs/WDRs hereafter), 24-hour recall, food frequency questionnaires, diet history, replicate food methods and biochemical analysis^1^^,^^2^^)^. Each procedure has its own strengths and weaknesses in terms of elements of diet, time frame and the scope of the study. Some are adequate for assessing nutrient intake in the population and can be employed for ecological studies, while others are more appropriate for ranking/categorizing subjects for epidemiological research, including cohort and case-control studies.

Among available approaches, DRs/WDRs appear the most precise and are often used for quantifying consumption of foods/nutrients in defined populations and ascertaining population means. Accordingly, there are many articles reporting intake of foods and nutrients using DRs/WDRs^1^^,^^2^^)^. Multiple/long-term data are accepted as *gold standards/references* for dietary surveys including in Japan^3^^)^. We have also carried out a relative validation study and observed satisfactorily higher relative validity of a semi-quantitative food frequency questionnaire (abbreviated SQFFQ hereafter) versus four season consecutive 7 day WDRs (28 day WDRs) in Japanese dietitians, as reported previously^4^^,^^5^^)^.

Although information from DRs/WDRs seems accurate on the actual day of food intake, as is reported^1^^,^^2^^)^, there are variances in dietary consumption according to day, week, season and person, and many days are needed to estimate habitual intake of foods and nutrients. Here we examined intake of energy and 30 nutrients and relative magnitude of daily, weekly, seasonal, within/intra- and between/inter-individual variance, and calculated the number of days needed to evaluate an individual’s nutrient intake within 10% and 20% of the true mean with a specified degree of the error on the basis of four season consecutive 7 day WDRs provided by 80 Japanese female dietitians. Few studies on daily, weekly and seasonal fluctuations on intake of foods and nutrients have been conducted in Japanese population. Furthermore, dietitians are professionals of diet and health, and information on consumption of selected nutrients may naturally be assumed to be accurate/reliable. Little research, however, has been carried out employing dietitians as study subjects, not only in Japan but also in other areas of the world.

## SUBJECTS AND METHODS

### Subjects and 28 day WDRs

We earlier evolved an evidence-based SQFFQ according to multiple regression and contribution analyses, and validated the SQFFQ against 28 day WDRs, as described elsewhere^4^^,^^5^^)^. This study was based on the same 28 day WDRs or four season consecutive 7day WDRs. Briefly, in autumn 1996, we first mail-administered the SQFFQ to 106 (21 male and 85 female) middle-aged Japanese dietitians living in Aichi Prefecture, in Central Japan and self-administered consecutive 7 day WDRs approximately a week later, and at about 3 month intervals in winter, spring and summer 1997.

Eighty female dietitians completed the four season consecutive 7 day WDRs. Male dietitians were excluded because they were rather small in number. This investigation was therefore based on the 28 day WDRs provided by eighty female dietitians with a mean age (years of age) ± standard deviation (minimum - maximum) of 48 ± 8 (32 - 66) in autumn, 1996. The values for height (cm), weight (kg) and BMI (kg/m^2^) were 156.9 ± 5.1 (146.1 - 172.1), 52.2 ± 5.5 (37.5 - 69.4), and 21.5 ± 2.2 (15.9 - 29.5), respectively.

Food weight was quantified before cooking if it was prepared at home, otherwise after cooking. Foods were generally weighed individually; while those like soup/stew were divided by the number of household members. For foods eaten out, reference was made to model recipes. For immeasurable foods, standard portion size was applied. Although WDRs were satisfactorily filled out, research nutritionists reviewed their accuracy as well as completeness.

### Nutrients Selected

We chose energy and 30 nutrients which include protein, fat, carbohydrate, vitamins (including carotenes, and vitamins A, D, E and C), minerals (including potassium, calcium, magnesium, phosphorus, iron, zinc and copper) and total dietary fiber (TDF) (including soluble DF and insoluble DF). Fat was divided into saturated fatty acids (abbreviated SFAs hereafter) (including myristic acid (14:0), palmitic acid (16:0), and stearic acid (18:0)), monounsaturated fatty acids (abbreviated MUFAs hereafter) (including palmitoleic acid (16:1) and oleic acid (18:1)), polyunsaturated fatty acids (abbreviated PUFAs hereafter), n-6 PUFAs, n-3 PUFAs and cholesterol, n-6 PUFAs were separated into linoleic acid (18: 2n-6) and arachidonic acid (20: 4n-6), and n-3 PUFAs into *α*-linolenic acid (18: 3n-3), eicosapentaenoic acid (EPA, 20: 5n-3) and docosahexaenoic acid (DHA, 22: 6n-3).

### Calculation of Intake of Energy and Nutrients

We computed average daily consumption of major and micro-nutrients by multiplying the food intake (in grams) or serving/portion size and the nutrient content per 100 grams of food as listed in the Standard Tables of Food Composition, Version 4, the Follow-up of Standard Tables of Food Composition^6^^,^^7^^)^, Composition Table of Processed Foodstuff^8^^)^ and Table of Trace Element Contents in Japanese Foodstuff^9^^)^. Since the composition tables are incomplete for certain foods, estimation was largely made according to analogies from same/similar foods and to assessment based on model recipes for relevant foods as reported^10^^)^.

### Statistical Analysis

Using all data of 2,240 day WDRs or four season consecutive 7 day WDRs completed by 80 female dietitians, we firstly calculated mean and standard deviation along with minimum and maximum values, and compared differences in intakes of selected nutrients by day, week, season and person according to analysis of variance (ANOVA)^11^^)^.

We examined the relative contributions of daily, weekly, seasonal, between-individual variation and residual to total variation according to analysis of components of variance^11^^-^^13^^)^.

The ratio of within- and between-individual variance with 95% confidence intervals was then computed^11^^-^^13^^)^. In this calculation, variation due to day, week and season was included in within-individual variation. Furthermore, we quantified minimal days required to estimate nutrient consumption within 10% and 20% of the true mean with 95% confidence intervals on the basis of within-individual variance^11^^-^^13^^)^.

## RESULTS

### Intake of Energy and Nutrients

Average and standard deviation (minimum-maximum) of daily intakes of energy and 30 nutrients are shown in Table 1. Mean daily intakes of selected nutrients by season are given in Table 2. Most nutrients, except for carbohydrate, phosphorus, vitamin D, oleic acid, *α*-linolenic acid and cholesterol, were significantly different by season. Remarkable seasonal differences were observed for carotenes, vitamin A, vitamin C, iron, zinc, AA, n-3 PUFAs, EPA, DHA and dietary fiber, including TDF, SDF and IDF (p<0.001). Among these, daily intakes of carotenes and vitamin C in autumn were about 20% larger than in summer. Table 3 shows results of ANOVA for intake of energy and 30 nutrients. Intakes of several nutrients were prevalently influenced by the sequence of days but not by day of week. Consumption of energy and carbohydrate, for instance, was larger in weekend than weekday (data not shown). Intake of energy and all nutrients significantly differed by person (p<0.001 for all).

**Table 1.** Average daily intake of energy and 30 nutrients according to 28 day WDRs.

Nutrient		Mean	SD	Minimum	Maximum
Energy	[ kcal ]	1,820	352	292	3,450
Protein	[ g ]	74.3	17.3	8.5	176.5
Fat	[ g ]	56.3	18.9	6.2	160.1
Carbohydrate	[g ]	243.6	52.5	51.1	483.8

Carotenes	[ µg ]	3,620	2,430	11	16,015
Vitamin A	[ µgRE^1)^ ]	756	693	29	9,310
Vitamin D	[ µg ]	7.4	9.0	0	71.4
Vitamin E	[ mg *α*-TE^2)^ ]	8.9	3.2	0.6	32.2
Vitamin C	[ mg ]	144	78	6	758

Potassium (K)	[ mg ]	2,913	785	613	6,315
Calcium (Ca)	[ mg ]	632	249	107	2,361
Magnesium (Mg)	[ mg ]	255	73	21	718
Phosphorus (P)	[ mg ]	1,076	259	215	2,481
Iron (Fe)	[ mg ]	10.8	3.2	1.0	27.4
Zinc (Zn)	[ mg ]	8.5	4.8	0.6	72.2
Copper (Cu)	[ mg ]	1.2	0.5	0.1	7.2

SFAs^3)^	[ g ]	15.6	6.5	2.1	57.0
MUFAs^4)^	[ g ]	19.1	7.7	1.6	57.0
Oleic acid	[ g ]	16.7	6.8	1.4	47.4
PUFAs^5)^	[ g ]	13.1	5.2	0.4	41.4

n-6PUFAs	[ mg ]	10,622	4,453	379	36,680
Linoleic acid	[ mg ]	10,433	4,433	374	36,168
Arachidonic acid	[ mg ]	136	73	1	515

n-3PUFAs	[ mg ]	2,495	1,363	31	10,704
*α*-Linolenic acid	[ mg ]	1,550	838	23	6,398
EPA^6)^	[ mg ]	274	360	0	2,865
DHA^7)^	[ mg ]	509	529	0	4,631

Cholesterol	[ mg ]	365	192	0	1,907

TDF^8)^	[ g ]	15.7	5.4	2.1	41.4
SDF^9)^	[ g ]	3.0	1.5	0.1	54.2
IDF^10)^	[ g ]	1.9	4.1	1.5	55.2

^1)^ RE : Retinol equivalent ^2)^ TE : Tocopherol equivalent

^3)^ SFAs: Saturated fatty acids ^4)^ MUFAs: Monounsaturated fatty acids

^5)^ PUFAs: Polyunsaturated fatty acids ^6)^ EPA: Eicosapentaenoic acid

^7)^ DHA: Docosahexaenoic acid ^8)^ TDF: Total dietary fiber

^9)^ SDF: Soluble dietary fiber ^10)^ IDF: Insoluble dietary fiber

**Table 2.** Average daily intake of energy and 30 nutrients by season.

Nutrient		October 1996		January 1997		April 1997		August 1997		
		(Autumn)		(Winter)		(Spring)		(Summer)		
		Mean	SD	Mean	SD	Mean	SD	Mean	SD	
Energy	[ kcal ]	1,852	346	1,825	352	1,811	348	1,792	362	**
Protein	[ g ]	75.7	15.9	74.8	17.3	73.6	17.9	73.1	18.1	**
Fat	[ g ]	58.7	19.1	55.5	18.5	55.9	18.6	54.9	19.4	***
Carbohydrate	[ g ]	245.5	51.6	245.9	52.3	242.8	53.2	240.1	52.8	

Carotenes	[ µg ]	3,975	2,529	3,930	2,456	3,376	2,414	3,199	2,225	***
Vitamin A	[ µgRE ]	824	807	779	627	715	677	707	642	**
Vitamin D	[ µg ]	7.8	9.3	7.4	9.4	7.4	8.6	6.9	8.6	
Vitamin E	[ mg *α*-TE]	9.2	3.4	8.6	3.1	8.7	3.1	9.0	3.2	***
Vitamin C	[ mg ]	160	82	154	74	136	72	128	78	***

Potassium (K)	[ mg ]	3,056	763	2,969	780	2,810	795	2,817	775	***
Calcium (Ca)	[ mg ]	662	250	642	257	602	239	623	245	***
Magnesium (Mg)	[ mg ]	264	72	258	73	248	74	249	73	***
Phosphorus (P)	[ mg ]	1,096	237	1,077	262	1,063	262	1,070	274	
Iron (Fe)	[ mg ]	11.4	3.3	11.2	3.2	10.6	3.2	10.1	2.9	***
Zinc (Zn)	[ mg ]	8.7	5.1	9.5	7.5	7.8	2.1	7.8	2.2	***
Copper (Cu)	[ mg ]	1.3	0.6	1.3	0.7	1.2	0.4	1.1	0.3	***

SFAs	[ g ]	16.4	6.9	15.6	6.6	15.3	6.0	15.1	6.6	**
MUFAs	[ g ]	19.7	7.6	18.9	7.6	19.1	7.6	18.7	7.7	*
Oleic acid	[ g ]	17.2	5.8	16.5	6.6	16.7	6.8	16.4	7.0	
PUFAs	[ g ]	13.8	5.4	12.7	4.9	13.1	5.1	13.0	5.2	**

n-6PUFAs	[ mg ]	11,046	4,767	10,232	4,113	10,687	4,411	10,563	4,502	**
Linoleic acid	[ mg ]	10,838	4,719	10,045	4,099	10,500	4,388	10,349	4,480	**
Arachidonic acid	[ mg ]	148	72	136	72	134	72	125	74	***

n-3PUFAs	[ mg ]	2,701	1,427	2,479	1,385	2,410	1,257	2,388	1,360	***
*α*-Linolenic acid	[ mg ]	1,588	807	1,519	814	1,558	866	1,536	863	
EPA	[ mg ]	327	396	283	373	243	312	245	347	***
DHA	[ mg ]	588	580	518	546	471	461	458	512	***

Cholesterol	[ mg ]	379	179	357	192	372	191	354	206	

TDF	[ g ]	16.4	5.4	16.5	5.5	15.1	5.4	14.7	5.3	***
SDF	[ g ]	3.2	1.5	3.3	1.1	2.7	1.4	3.0	1.5	***
IDF	[ g ]	12.5	5.4	12.5	4.2	11.7	4.0	11.1	3.9	***

*p<0.05, ** p<0.01, and ***p<0.001 according to ANOVA.

**Table 3.** Results of analysis of variance for intake of energy and 30 nutrients.

Nutrient	Sequence of days	Day of week	Season	Person
Energy	*		**	***
Protein			**	***
Fat	*		***	***
Carbohydrate	**	**		***

Carotenes	***		***	***
Vitamin A			**	***
Vitamin D				***
Vitamin E	*		***	***
Vitamin C	***		***	***

Potassium (K)	***		***	***
Calcium (Ca)	**		***	***
Magnesium (Mg)	*		***	***
Phosphorus (P)				***
Iron (Fe)	***		***	***
Zinc (Zn)	***		***	***
Copper (Cu)	***		***	***

SFAs	*		**	***
MUFAs			*	***
Oleic acid				***
PUFAs	*		**	***

n-6PUFAs	*		**	***
Linoleic acid	*		**	***
Arachidonic acid	***		***	***

n-3PUFAs	**		***	***
*α*-Linolenic acid				***
EPA	***		***	***
DHA	**		***	***

Cholesterol				***

TDF	***		***	***
SDF	***		***	***
IDF	***		***	***

*p<0.05, ** p<0.01, and ***p<0.001 according to ANOVA.

### Relative Contributions of Daily, Weekly, Seasonal and Personal Variances

As shown in Table 4, the relative contributions of variance (%) by person were greatest for all nutrients. The contributions of residual variation of 52.3 - 91.1 were larger than between- individual variation of 6.9 - 45.3. The values for sequence by days, day of week, and by season ranged form 0.3 - 1.6, 0 - 0.3, and 0.1 - 2.7, respectively.

**Table 4.** Relative contributions of variance(%) for intake of energy and 30 nutrients.

Nutrient	Sequence of days	Day of week	Season	Between-individual	Residual
Energy	1.0	0.1	0.4	33.4	65.1
Protein	0.7	0.0	0.3	33.3	65.6
Fat	0.8	0.0	0.6	24.2	74.4
Carbohydrate	1.4	0.2	0.2	34.8	63.3

Carotenes	0.5	0.1	1.9	22.9	74.5
Vitamin A	0.7	0.0	0.5	9.8	89.1
Vitamin D	1.1	0.2	0.1	7.5	91.1
Vitamin E	1.0	0.0	0.6	19.7	78.7
Vitamin C	0.8	0.0	2.7	28.0	68.5

Potassium (K)	0.5	0.2	1.8	44.8	52.8
Calcium (Ca)	0.7	0.0	0.8	37.3	61.2
Magnesium (Mg)	0.3	0.0	0.8	39.9	58.9
Phosphorus (P)	0.6	0.0	0.2	37.9	61.3
Iron (Fe)	0.5	0.0	2.7	32.9	63.9
Zinc (Zn)	0.9	0.0	2.1	9.8	87.3
Copper (Cu)	0.7	0.0	2.0	14.1	83.3

SFAs	1.2	0.0	0.5	18.9	79.3
MUFAs	0.7	0.0	0.3	24.2	74.8
Oleic acid	0.7	0.0	0.2	23.8	75.2
PUFAs	1.0	0.1	0.6	22.8	75.6

n-6PUFAs	1.1	0.0	0.4	22.1	76.3
Linoleic acid	1.1	0.0	0.4	22.1	76.4
Arachidonic acid	1.3	0.0	1.2	15.5	81.9

n-3PUFAs	1.2	0.3	0.8	14.5	83.2
*α*-Linolenic acid	0.6	0.1	0.1	22.7	76.5
EPA	1.6	0.1	0.9	7.1	90.3
DHA	1.3	0.2	0.9	6.9	90.6

Cholesterol	0.7	0.0	0.3	18.1	80.9

TDF	0.3	0.0	2.1	45.3	52.3
SDF	0.4	0.0	2.5	33.2	63.9
IDF	0.4	0.1	2.1	44.8	52.6

### Comparison Between Within- and Between-individual Variances

Within-individual variances were larger than between-individual variances (Table 5), and the ratios were greater than one. They ranged from 1.3 (0.7 - 2.6) for potassium, TDF and IDF - 26.9 (15.1 - 53.0) for DHA. The ratios for micro-nutrients (1.3 - 26.9) were generally larger than those (2.1 - 3.6) for energy and major nutrients.

**Table 5.** Intake of energy and 30 nutrients, within- and between-individual variation, coefficients of variation and minimal days needed to estimate nutrient intake within 10% and 20% of the true mean with 95% confidence intervals.

	Mean		Sw^2^/Sb^2^	(95% CIs)		Coefficient of variation		Number of days needed to lie within 10% and 20% of true means					

						Within- individual	Between- individual	10%			20%		
								Mean	(95% CIs)		Mean	(95% CIs)	
Energy	1,820	[ kcal ]	2.2	( 1.2 -	4.3 )	16.1	10.8	10	( 10 -	11 )	3	( 3 -	3 )
Protein	74.3	[ g ]	2.2	( 1.2 -	4.3 )	19.4	13.1	15	( 14 -	16 )	4	( 4 -	4 )
Fat	56.3	[ g ]	3.6	( 2.0 -	7.2 )	29.8	15.6	35	( 32 -	37 )	9	( 8 -	10 )
Carbohydrate	243.6	[ g ]	2.1	( 1.2-	4.1 )	17.7	12.4	13	( 12 -	13 )	4	( 3 -	4 )

Carotenes	3,620	[ µg ]	3.9	( 2.2 -	7.7 )	60.0	30.3	139	( 122 -	160 )	35	( 31 -	40 )
Vitamin A	756	[ µgRE ]	14.2	( 8.0 -	28.0 )	88.6	23.5	302	( 273 -	337 )	76	( 69 -	85 )
Vitamin D	7.4	[ µg ]	23.1	( 13.0 -	45.5 )	119.6	24.9	550	( 494 -	617 )	138	( 124 -	155 )
Vitamin E	8.9	[ mg *α*-TE]	5.1	( 2.8 -	10.0 )	71.7	32.3	198	( 172 -	230 )	50	( 43 -	58 )
Vitamin C	144	[ mg ]	2.7	( 1.5 -	5.4 )	45.8	27.2	81	( 72 -	92 )	21	( 18 -	23 )

Potassium (K)	2,913	[ mg ]	1.3	( 0.7 -	2.6 )	20.4	17.7	16	( 15 -	18 )	4	( 4 -	5 )
Calcium (Ca)	632	[ mg ]	1.8	( 1.0 -	3.6 )	31.8	23.4	39	( 36 -	44 )	10	( 9 -	11 )
Magnesium (Mg)	255	[ mg ]	1.7	( 0.9 -	3.3 )	22.6	17.7	20	( 19 -	22 )	5	( 5 -	6 )
Phosphorus (P)	1,076	[ mg ]	1.8	( 1.0 -	3.5 )	19.3	14.5	15	( 14 -	16 )	4	( 4 -	4 )
Iron (Fe)	10.8	[ mg ]	2.3	( 1.3 -	4.4 )	25.0	16.7	25	( 23 -	26 )	7	( 6 -	7 )
Zinc (Zn)	8.5	[ mg ]	14.2	( 8.0 -	28.0 )	55.3	14.7	118	( 111 -	126 )	30	( 28 -	3 )
Copper (Cu)	1.2	[ mg ]	8.1	( 4.5 -	16.0 )	40.8	14.3	64	( 61 -	69 )	16	( 16 -	18 )

SFAs	15.6	[ g ]	5.3	( 3.0 -	10.5 )	38.5	16.7	57	( 53 -	62 )	15	( 14 -	16 )
MUFAs	19.1	[ g ]	3.6	( 2.0 -	7.0 )	35.6	18.3	49	( 45 -	53 )	13	( 12 -	14 )
Oleic acid	16.7	[ g ]	9.4	( 5.3 -	18.5 )	36.2	18.8	51	( 47 -	55 )	13	( 12 -	14 )
PUFAs	13.1	[ g ]	4.0	( 2.2 -	7.9 )	35.1	17.6	48	( 44 -	52 )	12	( 11 -	13 )

n-6PUFAs	10,622	[ mg ]	4.1	( 2.3 -	8.1 )	37.7	18.6	55	( 51 -	60 )	14	( 13 -	15 )
Linoleic acid	10,433	[ mg ]	1.6	( 0.9 -	3.2 )	38.2	18.7	57	( 52 -	62 )	15	( 13 -	16 )
Arachidonic acid	136	[ mg ]	6.8	( 3.8 -	13.5 )	50.1	20.2	97	( 89 -	106 )	25	( 23 -	27 )

n-3PUFAs	2,495	[ mg ]	7.7	( 4.3 -	15.1 )	51.2	18.5	101	( 93 -	110 )	26	( 24 -	28 )
*α*-Linolenic acid	1,550	[ mg ]	4.0	( 2.2 -	7.8 )	48.4	24.3	90	( 82 -	101 )	23	( 21 -	26 )
EPA	274	[ mg ]	25.4	( 14.2 -	50.0 )	129.0	25.5	640	( 573 -	720 )	160	( 144 -	180 )
DHA	509	[ mg ]	26.9	( 15.1 -	53.0 )	102.6	19.6	400	( 372 -	443 )	100	( 93 -	111 )

Cholesterol	365	[ mg ]	5.5	( 3.1 -	10.9 )	48.5	20.5	91	( 83 -	100 )	23	( 21 -	25 )

TDF	15.7	[ g ]	1.3	( 0.7 -	2.6 )	26.1	22.9	27	( 24 -	30 )	7	( 6 -	8 )
SDF	3.0	[ g ]	2.3	( 1.3 -	4.4 )	39.7	26.6	61	( 54 -	69 )	16	( 14 -	18 )
IDF	11.9	[ g ]	1.3	( 0.7 -	2.6 )	25.8	22.5	26	( 24 -	29 )	7	( 6 -	8 )

### Number of Days Needed to Evaluate the True Intake of Energy and Nutrients

Table 5 also shows coefficient of variance with 95% confidence intervals, and minimal days needed to ascertain an individual’s nutrient consumption within 10% (20%) of the true mean with 95% confidence intervals, which ranged from 15 - 640 (4 -160) for micro-nutrients, longer than those for energy and major nutrients which ranged from 10-35 (3 - 9).

## DISCUSSION

As reported in the literature^12^^,^^14^^-^^22^^)^, the greatest source of variation in nutrient intake was brought about by person. Within-individual variation was greater than between-individual variation, which may be largely thanks to our free-living lifestyle in the real world, far from data for basic sciences like chemistry and physics. The extent of within-individual variability would unduly yield misclassification of subjects and inconsistencies in epidemiological observations^1^^,^^2^^)^. Every effort should therefore be exerted to evade categorization bias in studies related to dietary surveys in addition to statistical procedures for adjusting within-individual fluctuation.

When assessing within-individual variation and the ratio of within- vs. between-individual variance, the settings for DRs/WDRs, including age and sex of the subjects, number of days, and study frames (food vs. recipe based DRs/WDRs), should clearly be taken into account. Those values for most nutrients in this study were generally similar to those measured in the general population in Japan^20^^,^^21^^)^; however, in common, they were greater than those calculated from DRs/WDRs in western countries^14^^-^^19^^)^, which may be partly due to the wide variety of the Japanese diet.

The next largest variation was derived from season^23^^-^^28^^)^. For this reason, we should keep in mind to seasonal effects in dietary studies. For example, intakes of certain vitamins and minerals in autumn and winter were found to be larger than those in spring and summer, which may be associated with fluctuations in consumption of fruit, vegetables and certain seafood because they are important sources of relevant micronutrients. Contrary to the comparison based on absolute values, the relative contribution of variance by season to the total variance were not so large as expected because the consumption of fruit and vegetables may be evenly increased in the study subjects in autumn and winter.

There were differences in intakes of selected nutrients by sequence of days but not by day of week as reported in the literature^12^^,^^14^^,^^19^^)^. The difference by day of week was greatest for carbohydrate in this study (p<0.001), rather than sequence of days, because bigger meals were usually consumed on the weekend than on weekdays. Therefore, the information of dietary intake during weekend should not be overlooked in dietary surveys.

With our WDRs, minimal days required for evaluating a person’s true intake of energy and macro-nutrients were generally fewer than for micro-nutrients in line with earlier findings^12^^,^^18^^,^^21^^,^^22^^)^. At least 10 days were needed for energy to secure values within 10% of the true mean, in contrast to one year or more for vitamin A, vitamin D, EPA and DHA. Accordingly, we should always remember that short-term DRs/WDRs invariably yield invalid/inconsistent information, when assessing the association between consumption of micro-nutrients, in particular, and health/disease.

The accuracy of DRs/WDRs is, as mentioned, greatest when taken on the actual day of ingestion of foods/nutrients. DRs/WDRs have been adopted for nutritional surveys in the world including Japan and used for estimating the population mean. They are employed as *gold standards/references* for dietary surveys; however, diet varies according to day, week, season and person. In order to precisely assess average individual habitual consumption of foods/nutrients, especially for certain micro-nutrients, we need long-term DRs/WDRs due to within-individual variation as described above. It is obviously very laborious to keep multiple DRs/WDRs. Thus, it does not seem pertinent to administer multiple DRs/WDRs, particularly to the general populace, but rather apt for FFQs/SQFFQs instead to ascertain long-term dietary intake.

Although less precise/less quantifiable than DRs/WDRs, FFQs/SQFFQs may be suitable for case-referent and prospective studies. In this sense, we developed a data-based SQFFQ and examined relative validity of the SQFFQ versus 28 day WDRs as well as its reproducibility. Using the validated/modified SQFFQ, we proposed the JADE (Japanese Dietitians’ Epidemiologic) Study and self-administered SQFFQ to Japanese dietitians in autumn 1999, 2000 and 2001, which is worldwide a new approach, to elucidate relationships between diet/lifestyle and health/disease.
